# Separate double-layer repair versus en masse repair for delaminated rotator cuff tears: a systematic review and meta-analysis

**DOI:** 10.1186/s13018-020-01689-4

**Published:** 2020-05-13

**Authors:** Jia Chen, Zhen-Yang Zheng, Yi-Ming Ren

**Affiliations:** 1 Department of Traumatic Orthopedics, Tianjin 4th Central Hospital, Tianjin, PR China; 2 Department of Joint and Sport Medicine, Tianjin Union Medical Center, Tianjin, PR China; 3 Tianjin, 300143 PR China

## Abstract

**Objective:**

Delaminated rotator cuff tears are a common shoulder disorder in elderly individuals. Either arthroscopic separate double-layer repair (DR) or en masse repair (ER) is used to treat a delaminated rotator cuff tear. We conducted this systematic review and meta-analysis to compare the clinical outcomes of arthroscopic ER versus DR intervention.

**Methods:**

Five studies were acquired from PubMed, Medline, Embase, CNKI, Google, and the Cochrane Library. The data were extracted by two of the coauthors independently and were analyzed with RevMan 5.3. Mean differences (MDs), odds ratios (ORs), and 95% confidence intervals (CIs) were calculated. The Cochrane Collaboration’s risk of bias tool and Newcastle–Ottawa Scale were used to assess the risk of bias.

**Results:**

Five studies, including two randomized controlled trials (RCTs) and three observational studies, were assessed. The methodological quality of the trials ranged from low to high. The pooled results for the Shoulder Rating Scale of the University of California at Los Angeles (UCLA) score, visual analog scale (VAS) score, Constant score, and range of motion (ROM) showed that the outcomes were not statistically significant between the two interventions. The difference in retear rate was not statistically significant (OR = 0.69, 95% CI = 0.36–1.33, *P* = 0.27). The sensitivity analysis proved the stability of the pooled results, and publication bias was not apparent.

**Conclusions:**

Both arthroscopic ER and DR interventions had benefits in delaminated rotator cuff tear treatment. ER and DR treatments were equally effective and had the same retear rate. The arthroscopic DR technique could not be recommended as the optical choice for delaminated rotator cuff tears based on current evidence.

## Introduction

Rotator cuff tears are a common injury of the shoulder joint, which limit the movement of the shoulder joint and, at worst, seriously affects some patients’ daily life. The incidence is 5–40% [1], and 38–92% of patients have a transverse tear larger than 5 mm between the bursa side and the articular side tendon during microscopic exploration, which is called rotator cuff delamination tear and is often ignored in clinical treatment [2]. Through biomechanics analysis, it was found that the reason for the delamination of rotator cuff tears is that there is shear force between the two layers [3], and the articular layer is easier to retract after rotator cuff injury than the bursal layer [4], which produces greater tension and even repositioning difficulty during the reduction, increasing the difficulty of rotator cuff tissue reduction and the risk of the rotator cuff retearing after the operation. The main purpose of arthroscopic repair of a rotator cuff laceration is to relieve the patients’ pain, restore the function of the shoulder joint, and restore the anatomical structure of the rotator cuff as much as possible.

Previous studies have shown that delaminated rotator cuff tears have a serious negative impact on the healing of rotator cuff tissue and long-term functional recovery [5]. In the traditional operation method, the layered repair of the rotator cuff is based on the initial internal and external clinical anatomical structure of the rotator cuff tendon, and its practical clinical significance needs to be further explored [6]. Full-thickness repair is the most commonly used operation method for small rotator cuff tears, as the operation is easy and has a clinical therapeutic effect [7–9]. However, with the further accumulation of clinical experience, we found that for large and medium-sized rotator cuff delamination tears, the rotator cuff articular layer is often difficult to reset during the operation, and the abovementioned operation methods are difficult. Forced traction reduction may increase the risk of retearing after the operation, leading to failure of the operation and an increase in the pain of the patients.

To provide more evidence for clinical decisions, we conducted this systematic review and meta-analysis with related randomized controlled trial (RCT) studies and observational studies to compare the efficacy and occurrence of retearing in separate double-layer repair (DR) versus en masse repair (ER) for delaminated rotator cuff tears.

## Materials and methods

Ethical approval and patient consent were not required since the present study was a review of previously published literature.

### Inclusion criteria for published studies

#### Types of studies

We considered all published and unpublished RCTs and observational studies, including retrospective and prospective studies.

#### Types of participants

Patients were included only if they had a medium to large (tear size < 5 cm) full-thickness supraspinatus tear with separation (delamination) of the bursal and articular layers of the torn tendon. A tear was considered delaminated if the torn edge and cleavage tearing was ≥ 5 mm. No further subclassification of the degree of delamination was attempted. Lesions were confirmed by using magnetic resonance imaging (MRI) before surgery and arthroscopic visual inspection at the time of repair. To be included in the study, the tears had to be degenerative without any evident trauma history. Exclusion criteria included patients with massive rotator cuff tears, a history of shoulder surgery, or concomitant shoulder stiffness or lesions, such as arthritis in the glenohumeral joint or labral lesions.

#### Types of interventions

All surgical techniques including the arthroscopic DR and ER suture-bridging and double-row techniques were considered. The exclusion criteria were as follows: (1) insufficient clinical outcome data in the studies and (2) reviews, letters, or conference articles.

#### Types of outcome measures

The primary outcome measures were the clinical outcomes synthesizing the American Shoulder and Elbow Surgeons (ASES) score, Simple Shoulder Test (SST) score, Constant score, the Shoulder Rating Scale of the University of California at Los Angeles (UCLA) score, and the visual analog scale (VAS) score. The secondary outcomes included (1) postoperative active range of motion (ROM) (forward flexion, external rotation) and (2) the retear rate.

### Search methods for identification of studies

Six databases (PubMed, Medline, Embase, CNKI, Google, and the Cochrane Library) were searched using keywords such as “rotator cuff tear or rotator cuff injuries or rotator cuff tear arthropathy,” “delaminated or delamination,” “double-row or double row,” “suture-bridge or suture-bridging,” “surgery or surgical or operation,” and “arthroscopic or arthroscopy” through January 2020 to collect relevant studies containing clinical comparisons of DR versus ER for delaminated rotator cuff tears. The titles and abstracts of potential related articles identified by the electronic search were reviewed. References from retrieved articles were also assessed to extend the search strategy.

### Data collection and quality assessment

Two authors (YMR and ZYZ) independently assessed the titles and abstracts of all the studies screened during the initial search, and they excluded any clearly irrelevant studies using the inclusion criteria. Data were independently extracted using a standard data form for the first author’s name, year of publication, sample size, sex, age, intervention, country, study design, follow-up, and relevant outcome. A third author (JC) handled any disagreement about the inclusion of a study and helped reach a consensus. The Cochrane Collaboration’s risk of bias tool [10] was manipulated for the appraisal of RCT study quality. Observational studies were assessed by the Newcastle–Ottawa Scale, which includes 8 items [11]. A higher overall score indicates a lower risk of bias, and a score of 5 or less (out of 9) corresponds to a high risk of bias.

### Statistical analysis

RevMan statistical software v5.3 was used for the meta-analysis. The analysis of continuous variables was conducted with mean differences (MDs) and 95% confidence intervals (CIs). For a dichotomous outcome, we calculated the odds ratios (ORs) and 95% CIs. Heterogeneity was assessed by chi-squared and *I*^2^ tests. *P* < 0.05 and *I*^2^ > 50% indicated significant heterogeneity, and random-effect models were applied. Otherwise, fixed-effect models were used if there was no significant heterogeneity (*P* ≥ 0.05, *I*^2^ ≤ 50%). Sensitivity analyses were performed by omitting one study at a time to determine the stability of the pooled results. Publication bias was determined by a funnel plot.

## Results

### Study identification and inclusion

**Fig. 1:**
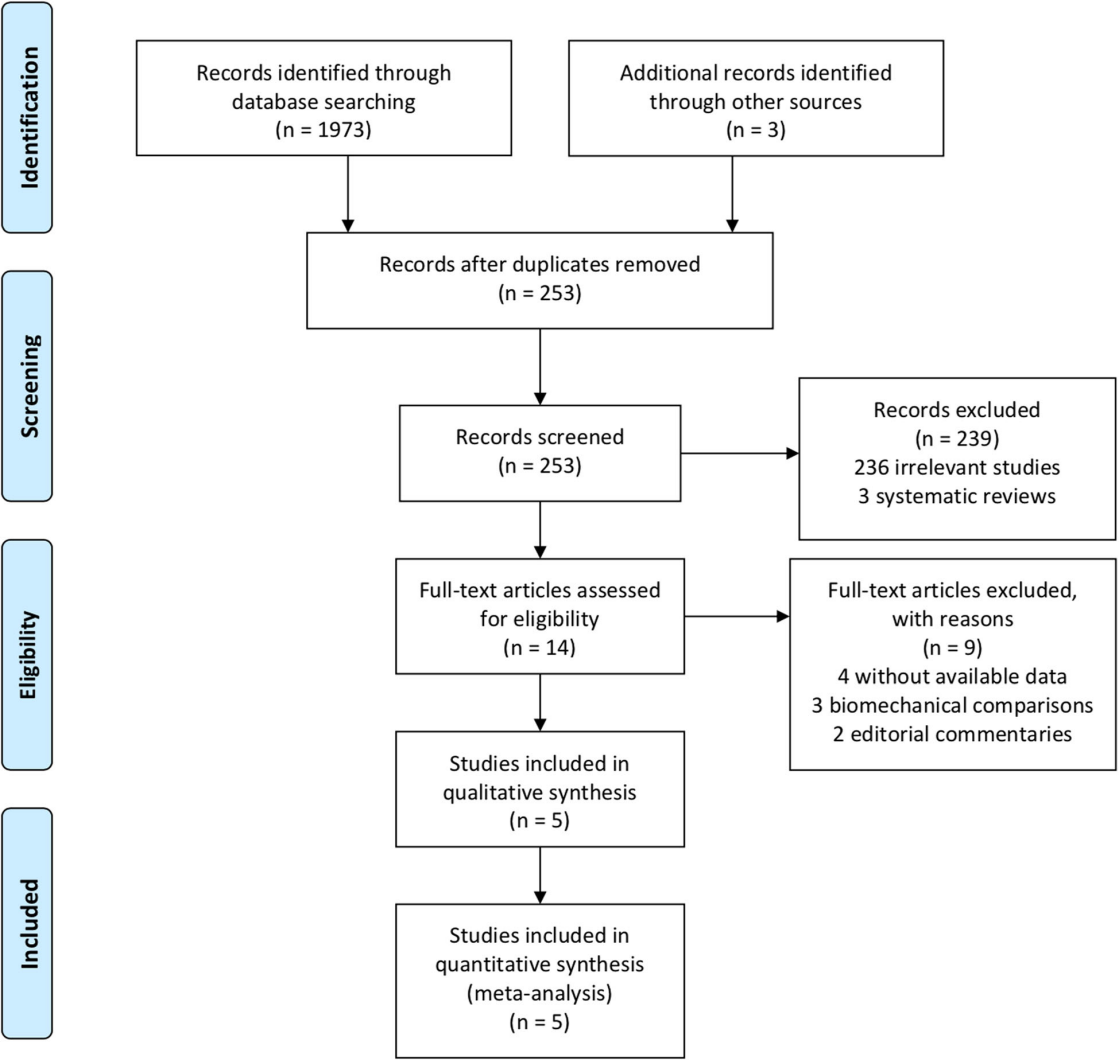
PRISMA flow diagram

Searches conducted in the PubMed, Medline, Embase, CNKI, Google, and Cochrane Library databases and other sources yielded a total of 1976 articles. After removing duplicates, 253 studies remained. Based on the title and abstract review, 236 irrelevant articles, 3 of which were systematic reviews, were excluded. Fourteen full-text articles were assessed for eligibility. However, nine articles were excluded based on the previously established exclusion criteria (4 without available data, 3 biomechanical comparisons, and 2 editorial commentaries). Finally, five trials (two RCTs and three observational studies) were included in this systematic review and meta-analysis. The flow diagram and PRISMA checklist are included in Fig. 1 and an Additional file 1.

### Study characteristics

We assessed 5 studies [12–16] including 2 RCTs and 3 retrospective studies in this article. The included studies were conducted in 3 countries (Japan, Korea, and China) from 2016 to 2019 and involved 255 patients (165 patients treated with the ER technique and 190 patients treated with the DR technique) aged 52.1 to 65.5 years. The average follow-up duration ranged from 12 to 29 months. The clinical outcomes of the studies were evaluated mainly based on the Constant score, UCLA score, VAS score, ROM, and retear rate. The detailed information of the included studies is shown in Table 1.

**Table 1:** Characteristics of studies included

	Year	Sample size (ER/DR)	Female (%)	Mean age (years)	Intervention		Country	Study design	Follow-up (month)	Relevant outcome
					ER	DR				
Cha et al [12]	2016	11/53	39.1%	ER63.7 ± 8.4 DR62.5 ± 7.6	Single-layer repair	Dual-layer double-row repair or dual-layer suture bridge repair	Korea	Retrospective study	ER28.1 ± 10.5 DR26.2 ± 9.7	VAS score; constant score; UCLA score; postoperative range of motion; retear rate
Kim et al [13]	2016	48/34	67.1%	ER65.2 (45–76) DR65.5 (47–78)	Conventional en masse repair	Separate double-layer double-row repair	Korea	RCT study	ER25.8 ± 1.5 DR25.9 ± 2.2	VAS score; constant score; ASES score; SST score; postoperative range of motion; retear rate
Ren et al [14]	2017	26/28	57.4%	ER55.3 ± 8.4 DR53.6 ± 6.1	Whole-layer repair	Separate double-layer repair	China	RCT study	12	VAS score; constant score; ASES score; UCLA score; postoperative range of motion; retear rate
﻿Nakamizo et al [15]	2018	52/46	51.0%	ER65.8 ± 8.5 DR64.1 ± 9.4	En masse suture-bridging	Dual-layer suture-bridging	Japan	Retrospective study	ER29.0 ± 9.1 DR27.6 ± 3.3	VAS score; UCLA score; SST score; postoperative range of motion; retear rate
Jia et al [16]	2019	28/29	42.1%	ER54.0 ± 8.3 DR52.1 ± 7.5	Whole-layer repair	Dual-layer suture-bridging	China	Retrospective study	23.3 (21–24)	VAS score; UCLA score; ASES score; constant score; postoperative range of motion; retear rate

*ER* en masse repair, *DR* double-layer repair, *RCT* randomized controlled trial, *VAS* visual analog scale, *ASES* the American Shoulder and Elbow, Surgeons, *UCLA* the Shoulder Rating Scale of the University of California at Los Angeles, *SST* Simple Shoulder Test

### Methodological assessment of study quality

**Fig. 2:**
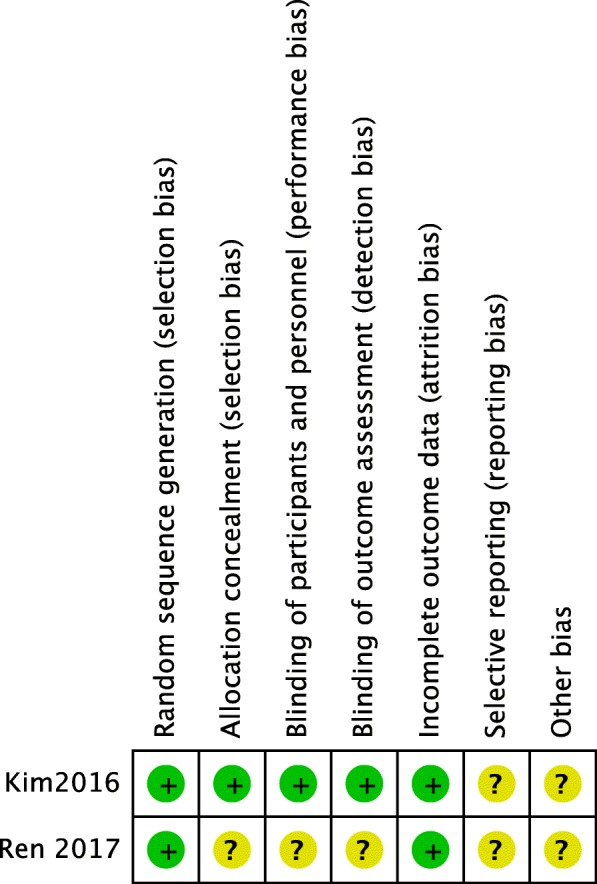
Risk of bias summary: this risk of bias tool incorporates the assessment of randomization (sequence generation and allocation concealment), blinding (participants and outcome assessors), incomplete outcome data, selective outcome reporting, and other risk of bias. The items were judged as “low risk,” “unclear risk,” or “high risk.” Green means “low risk,” red means “high risk,” and yellow means “unclear risk”

The methodological quality assessment of the five included studies is presented in Fig. 2 and Table 2. Among the RCTs, Ren’s study [14] clearly described the random sequence generation by random number tables, but blinding and allocation concealment were not mentioned, so it could be regarded as a low-quality study. Kim’s study [13] randomly assigned groups, had double-blind group assignments, and had double-blind assessments, and the group allocation was kept secret with a sealed envelope; this study was considered a high-quality study. Among the observational studies, the Newcastle–Ottawa Scale, including the exposed cohort, the non-exposed cohort, ascertainment of exposure, outcome of interest, comparability, assessment of outcome, length of follow-up, and adequacy of follow-up, was used to assess the risk of bias. The scores of all 3 studies were 8, indicating a low risk of bias.

**Table 2:** Methodological quality assessment

Study	Selection				Outcome				
	Exposed cohort	Non-exposed cohort	Ascertainment of exposure	Outcome of interest	Comparability	Assessment of outcome	Length of follow-up	Adequacy of follow-up	Total score
Cha et al. [12]	*	*	*	*	*	*	*	*	8
Nakamizo et al. [15]	*	*	*	*	*	*	*	*	8
Jia et al. [16]	*	*	*	*	*	*	*	*	8

*Risk of bias was assessed using the Newcastle–Ottawa Scale. A higher overall score indicates a lower risk of bias; a score of 5 or less (out of 9) corresponds to a high risk of bias

### Comparison of the constant scores for ER and DR

**Fig. 3:**
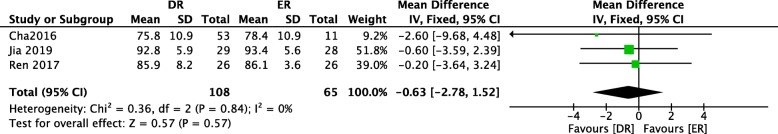
Forest plot of comparison: constant score between arthroscopic separate double-layer repair (DR) and en masse repair (ER) technique for delaminated rotator cuff tears

A comparison of postoperative constant scores for ER and DR was conducted among the 3 included studies [12, 15, 16], which included 173 patients (65 patients receiving ER and 108 patients receiving DR), as shown in Fig. 3. Heterogeneity testing showed that there was no heterogeneity among the studies (*P* = 0.84, *I*^2^ = 0%), so a fixed-effect model was used to pool the data from the 3 studies. The pooled results showed that the difference was not statistically significant between the ER group and the DR group (MD = − 0.63, 95% CI = − 2.78–1.52, *P* = 0.57).

### Comparison of the VAS scores for ER and DR

**Fig. 4:**
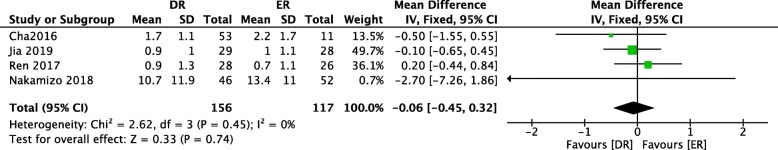
Forest plot of comparison: VAS score between arthroscopic separate double-layer repair (DR) and en masse repair (ER) technique for delaminated rotator cuff tears

A comparison of postoperative VAS scores for ER and DR treatment was conducted among 4 included studies [12, 14–16], which contained 273 patients, as shown in Fig. 4. A heterogeneity test showed that there was no heterogeneity among studies (*P* = 0.45, *I*^2^ = 0%), so a fixed-effect model was used. The overall estimate showed that the difference between the two groups was not statistically significant (MD = − 0.06, 95% CI = − 0.45–0.32, *P* = 0.74).

### Comparison of the UCLA scores for ER and DR

**Fig. 5:**
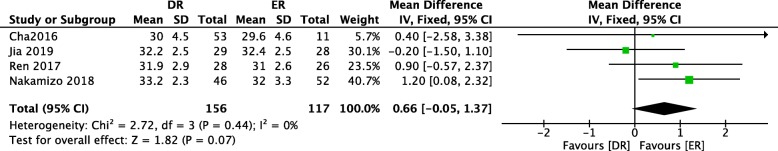
Forest plot of comparison: UCLA score between arthroscopic separate double-layer repair (DR) and en masse repair (ER) technique for delaminated rotator cuff tears

In Fig. 5, 4 included studies [12, 14–16] consisting of 273 patients (117 patients received ER treatment and 156 patients received DR treatment) investigated the postoperative UCLA score. No heterogeneity among studies (*P* = 0.44, *I*^2^ = 0%) was found, so we used a fixed-effect model to pool the data. The overall estimate showed that the difference was not statistically significant between the ER group and the DR group (MD = 0.66, 95% CI = − 0.05–1.37, *P* = 0.07).

### Comparison of ROM for ER and DR

**Fig. 6:**
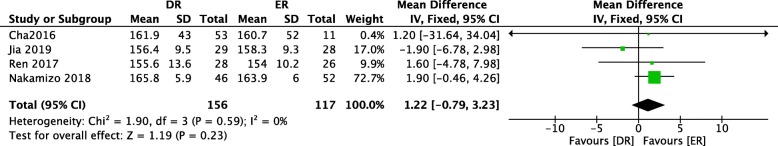
Forest plot of comparison: postoperative forward flexion between arthroscopic separate double-layer repair (DR) and en masse repair (ER) technique for delaminated rotator cuff tears

**Fig. 7:**
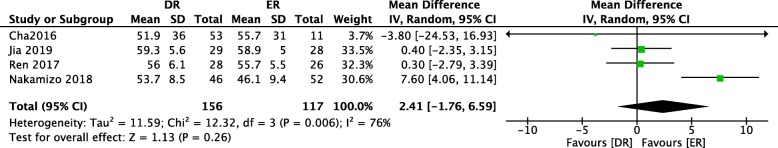
Forest plot of comparison: postoperative external rotation arthroscopic separate double-layer repair (DR) and en masse repair (ER) technique for delaminated rotator cuff tears

Four included studies [12, 14–16], including 117 ER surgery group cases and 156 DR surgery group cases, provided data for postoperative forward flexion. A heterogeneity test revealed that no heterogeneity existed among the studies (*P* = 0.59, *I*^2^ = 0%), and a fixed-effect model was used. A pooled analysis revealed that there was no significant difference between the ER surgery and DR surgery groups (MD = 1.22, 95% CI = − 0.79–3.23, *P* = 0.23) (Fig. 6). A comparison of postoperative external rotation for the two groups was conducted among 4 included studies [12, 14–16], which contained 273 patients (117 patients received ER surgery and 156 patients received DR surgery treatment), as shown in Fig. 7. High heterogeneity was found among studies (*P* = 0.006, *I*^2^ = 76%), so a random-effect model was used. The pooled results showed that the difference between the ER surgery and DR surgery groups was not statistically significant (MD = 2.41, 95% CI = − 1.76–6.59, P = 0.26).

### Comparison of the retear rates for ER and DR

**Fig. 8:**
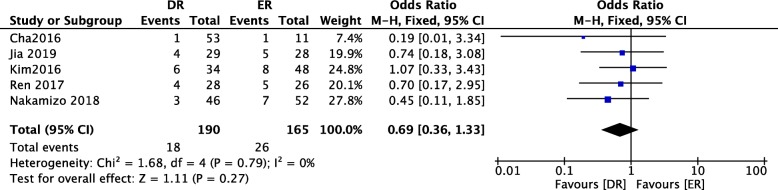
Forest plot of comparison: retear rate between arthroscopic separate double-layer repair (DR) and en masse repair (ER) technique for delaminated rotator cuff tears

In Fig. 8, five included studies [12–16], consisting of 355 delaminated rotator cuff tear patients (190 patients underwent DR and 165 patients underwent ER), reported the retear rate. No heterogeneity among studies (*P* = 0.79, *I*^2^ = 0%) was found, so we used a fixed-effect model. The overall estimate indicated that the pooled OR was 0.69 (95% CI = 0.36–1.33, *P* = 0.27), suggesting that the difference was not statistically significant between the DR intervention and the ER intervention.

### Sensitivity analysis and publication bias

**Fig. 9:**
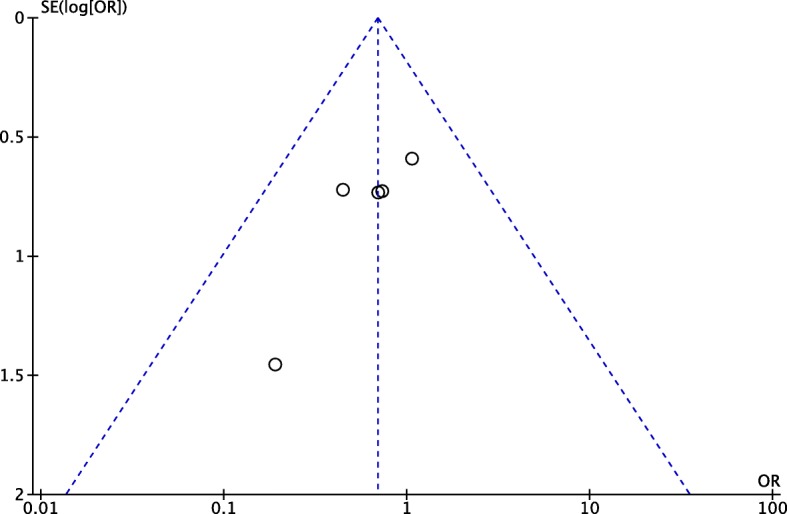
Funnel plot to test for publication bias. Each point represents a separate study for the indicated association. The vertical line represents the mean effects size. OR = odds ratio, SE = standard error

We performed a sensitivity analysis to assess the stability of the pooled results. For most outcome measures, the heterogeneity results were not obviously altered after sequentially omitting each study, indicating that our results were statistically reliable. The funnel plot of the included studies is shown in Fig. 9. The points in the funnel plot were almost symmetrically distributed, indicating that publication bias was not apparent.

## Discussion

### Summary of main results

In this study, we identified 2 RCTs and 3 observational studies to investigate the clinical outcomes of arthroscopic ER versus DR interventions. Our meta-analysis results showed that the outcomes were not statistically different between the two interventions in terms of UCLA score, constant score, VAS score, ROM, and retear rate. Kim’s RCT study results [13] showed that traditional ER and DR can significantly improve the clinical symptoms of patients with determined rotator cuff tears. Only the difference in VAS score was statistically significant (*P* < 0.05), and the pain relief obtained by layered repair was more obvious than that by full-thickness repair. Ren et al. thought that the operation time for layered suturing was longer than that for full-thickness suturing, and there was no significant difference in clinical effect [14]. In the study of Cha et al., the retear rate for ER was significantly higher than that for DR, and it was pointed out that the contraction direction of the two-layer structure after a layered tear of the rotator cuff was mainly in the posterior inner direction. When using the DR technology of double row anchor screws, the best anatomical balance could be achieved based on the contraction direction of each layer’s structure [15]. With further research on the anatomical structure of the footprint area of the rotator cuff, it was found that there are two different layers of tissue structure on the end of the footprint area of the rotator cuff and that the two layers of tissue structure have different tension. The rotator cuff tear often leads to a change in stratification, and each layer of structure has a different degree of retraction [12, 17]. Therefore, to restore the original anatomical structure of the rotator cuff in the footprint area, arthroscopic DR is increasingly used in clinical practice. Compared with traditional ER, arthroscopic-layered repair separately fixes the bursal side and articular side insertions of the torn rotator cuff, which conforms to the original anatomical structure of the rotator cuff and is more conducive to the healing of the torn rotator cuff than other repairs, in theory. Sugaya and other researchers mentioned that the articular side of a rotator cuff delamination tear originated from the oblique fiber behind the infraspinatus muscle, which was thicker than the transverse fiber originating from the combination of the supraspinatus muscle and infraspinatus muscle, and that the articular side had greater contraction tension than the bursal side, so DR could obtain better structural stability and functional recovery than traditional ER [18]. Mochizuki et al. proposed that in the two-layer structure of a rotator cuff delamination tear, the articular side is mainly composed of the articular capsule and stops at the inner side of the greater tuberosity, and the repair direction should be from the inner to the outer layer; the bursal side is mainly composed of infraspinatus muscle and stops at the front of the greater tuberosity, and the repair direction should be from the back to the front layer, so DR should be used for rotator cuff delamination tears [19]. Cheon et al. conducted a biomechanical study on the repair of delaminated rotator cuff tears in an animal model and found that separate layered repair was superior to en masse repair in the initial fixation strength but that 3 weeks later, in terms of histology and biomechanics, en masse suturing was superior to layered suturing [20]. Heuberer et al. proposed the double-layer cinch bridge, which had the advantages of reducing the effect of the suture knot on the microcirculation recovery of the determined rotator cuff, completing the anatomical reconstruction of the determined rotator cuff at the same time, and obtaining satisfactory clinical effects [21].

The retear rate in five included studies should also be discussed. Overall, 26 (15.8%) retears after undergoing ER surgery were reported, and 18 (9.5%) retears after undergoing DR surgery were reported in 5 of the included studies [12–16], which showed that DR has a lower retear rate than ER and is a better fixing technique. Twelve months after the operation in Ren’s study [14], 4 patients (14.3%) in the DR group and 5 patients (19.2%) in the ER group suffered from rotator cuff retearing, and there was no significant difference between groups (*χ*^2^ = 0.237, *P* = 0.626). In Nakamizo’s study [15], postoperative MRI showed Sugaya type IV or V discontinuity of the repaired tendons in 7 of 52 cases in the ER group and 3 of 46 cases in the DR group. The difference between the 2 groups was not significant (*P* = 0.327). In Jia’s study [16], 5 patients (17.9%) in the DR group and 4 patients (13.8%) in the ER group reported rotator cuff retearing, and there was no significant difference between groups (*χ*^2^ = 0.388, *P* = 0.824). Regarding rotator cuff integrity after repair according to the Sugaya classification, 8 patients in the ER group (17%) and 6 patients in the ER group (18%) had type 4 and 5 integrity (i.e., retears) (*P* > 0.05), 13 patients from the ER group (27%) and 9 patients from the DR group (27%) had type 3 integrity (i.e., insufficient thickness without discontinuity) (*P* > 0.05). No other complications related to the surgical procedures occurred in Kim’s study [13]. In Cha’s study, the follow-up MRI in the 26 cases in the DR group revealed 12 type I, six type II, six type III, and two type V results according to Sugaya’s classification [12]. There were two retears (7.6%), one in the dual-layer double-row group, and the other in the dual-layer suture bridge group. The follow-up MRI in the 11 cases of ER repair revealed five type I, three type II, and three (27.2%) type V results. A significant difference was observed between the two groups (*p* = 0.016). Three of the six cases that demonstrated Sugaya type III results were observed in the dual-layer double-row group, while the other three were observed in the dual-layer suture bridge group. Among the 18 with Sugaya type I and II structural integrity, two cases showed sustained delamination on MRI following surgery (11%), but the delamination disappeared in 16 cases (89%). Therefore, careful examination may be necessary for at least 6 months after rotator cuff repair for large or massive tears. Although the high rates of retears have been attributed to many factors, including the severity of the tear, tendon and bone quality, and muscle atrophy and fatty degeneration, repair techniques have been developed to improve the biomechanical properties of rotator cuff repair [18, 22–24].

### Limitations of the study

Some limitations of this study should be noted. First, the small sample size might have affected the significant difference between the two surgical procedures. Second, significant statistical heterogeneity of ROM still existed among the included trials, which may be explained by the clinical diversity among trials. Third, our study ignored the etiology of the disease, and further research is needed to discover whether these conclusions apply to patients with varying degrees and types of determined rotator cuff tears. Last but not the least, the included studies were mostly observational studies and not RCTs, and they largely relied on retrospectively collected data, resulting in a high risk of selection bias. More large-sample, multicenter, high-quality randomized controlled trials are needed to verify the outcomes of this meta-analysis.

## Conclusions

In conclusion, both arthroscopic ER and DR interventions have benefits in delaminated rotator cuff tears. ER and DR treatment were equally effective and had the same retear rate. In view of the heterogeneity and different follow-up times, whether these conclusions are applicable should be further determined in future studies.

## Supplementary information

**Additional file 1.** PRISMA checklist and flow diagram

## Data Availability

The present study was a review of previous published literatures.

## Abbreviations

RCT: Randomized controlled trial
DR: Double-layer repair
ER: En masse repair
MRI: Magnetic resonance imaging
ASES: American Shoulder and Elbow Surgeons
SST: Simple Shoulder Test
UCLA: Shoulder Rating Scale of the University of California at Los Angeles
VAS: Visual analog scale
ROM: Range of motion
MD: Mean difference
CI: Confidence interval
OR: Odds ratio
