# Supplementary material for: Separate double-layer repair versus en masse repair for delaminated rotator cuff tears: a systematic review and meta-analysis
Source: J Orthop Surg Res. 2020 May 13;15:171. doi: 10.1186/s13018-020-01689-4 (PMC7222332; doi:10.1186/s13018-020-01689-4)
Supplement: Supplementary file 1 — Additional file 1. PRISMA checklist and flow diagram [file 13018_2020_1689_MOESM1_ESM.docx]

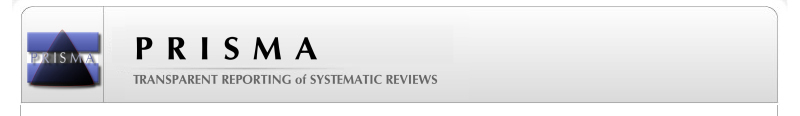
**PRISMA 2009 Flow Diagram**

Full-text articles excluded, with reasons
(n = 9)

4 without available data

3 biomechanical comparisons

2 editorial commentaries

Records excluded
(n = 239)
236 irrelevant studies
3 systematic reviews

Studies included in quantitative synthesis (meta-analysis)
(n = 5)

Studies included in qualitative synthesis
(n = 5)

Full-text articles assessed for eligibility
(n = 14)

Records screened
(n = 253)

Records after duplicates removed
(n = 253)

Additional records identified through other sources
(n = 3)

## Identification

## Eligibility

## Included

## Screening

Records identified through database searching
(n = 1973)
